# The Use of Positive Serological Tests as Evidence of Exposure to *Burkholderia pseudomallei*

**DOI:** 10.4269/ajtmh.2011.11-0114a

**Published:** 2011-06-01

**Authors:** Sharon J. Peacock, Allen C. Cheng, Bart J. Currie, David A. B. Dance

**Affiliations:** Department of Medicine; University of Cambridge; Cambridge, United Kingdom; E-mail: Sharon@tropmedres.ac; Department of Epidemiology and Preventive Medicine; Monash University; Melbourne, Australia;; Menzies School of Health Research, Darwin, Australia; Menzies School of Health Research and Northern Territory Clinical School; Royal Darwin Hospital; Darwin, Australia; Wellcome Trust-Mahosot Hospital-Oxford Tropical Medicine Research Collaboration; Microbiology Laboratory; Mahosot Hospital, Vientiane, Lao People's Democratic Republic; Centre for Clinical Vaccinology and Tropical Medicine, Churchill Hospital; University of Oxford, Oxford, United Kingdom

Dear Sir:

We note with interest Rolim and colleagues' cross-sectional serosurvey of residents of Tejuçuoca and Banabuiu in Ceará, Brazil to provide evidence of exposure to *Burkholderia pseudomallei*, the causative agent of melioidosis.^1^ Although there is definitive evidence of culture-confirmed melioidosis in that region,^2^ we caution against the use of an unvalidated enzyme-linked immunosorbent assay (ELISA) such as that used by Rolim and others to provide evidence of widespread exposure.

Although all serological tests are of relatively limited value for the diagnosis of melioidosis in patients living in areas where melioidosis is endemic (primarily because of seropositivity in the healthy population), the indirect hemagglutination assay (IHA) is widely accepted as the serological test of choice. The diagnostic performance of the IHA has been defined by several studies that used different cutoffs to take account of variable rates of background seropositivity. In the endemic region of Australia, the diagnostic sensitivity of an IHA titer of ≥ 1:40 was 56% at the time of admission (using culture as the gold standard), with evidence of subsequent seroconversion on serial IHA testing in 68% of patients who were initially IHA negative.^3^ In northeast Thailand, the diagnostic sensitivity of the much higher IHA titer of ≥ 1:160 was 72% and the specificity 64%.^4^ Other assays have been evaluated but have only been found to offer a marginal improvement on the IHA.^5^

Although the true incidence of culture-confirmed melioidosis in this region of Brazil is not known, the proportion of residents with positive serology in this study, reported as 58%, seems very high compared with known endemic areas where the incidence of confirmed melioidosis is known to be high. The sensitivity and specificity of the assay used by Rolim and others for indicating exposure to *B. pseudomallei* appears to be unknown from the data presented. One possible explanation for the high seropositivity observed is the use of an inappropriately low cutoff. We note that Rolim and colleagues also performed the ELISA used in their study on 20 serum samples from Australia that were negative by IHA, but were clinically uncharacterized, as was the negative control used in the test. Three of these samples were positive for immunoglobulin G (IgG) in their ELISA, of which one was also positive for IgM, suggesting a specificity for IgG of 85% (95% confidence interval [CI]: 62%, 97%) compared with IHA. A cut-off value was described as being the mean of the optical densities of a negative control. If the optical densities are normally distributed, it would then be expected that half the results from a negative control would be interpreted as positive. Another possible explanation for the high seropositivity observed is exposure to cross-reacting antigens in another environmental organism analogous to avirulent *Burkholderia thailandensis* as found in SE Asia.

We have previously raised concerns that apparently unvalidated assays are being used to provide evidence of exposure in other settings.^6^ Current recommendations for laboratory workers with exposure to *B. pseudomallei* suggest that baseline and post-exposure serology should be used for accurate interpretation of seropositivity after a potential exposure event, but the recommendations caution that a validated assay such as the IHA should be used.^7^ We have become aware that probable false positive results are occurring from at least one unvalidated serological assay, resulting in unnecessary anxiety in some laboratory workers with no evident exposure event.

We call for studies to develop and validate the use of a serological standard to assess exposure to *B. pseudomallei*. Ideally, such an assay should be accurate, inexpensive, simple to perform, and be reproducible between laboratories. In the interim, serological evidence of exposure should be based on assays with known sensitivity and specificity against culture-confirmed melioidosis.
